# Vibration from freight trains fragments sleep: A polysomnographic study

**DOI:** 10.1038/srep24717

**Published:** 2016-04-19

**Authors:** Michael G. Smith, Ilona Croy, Oscar Hammar, Kerstin Persson Waye

**Affiliations:** 1 Department of Occupational and Environmental Medicine, The Sahlgrenska Academy at the University of Gothenburg, Gothenburg, Sweden; 2 Department of Psychosomatic Medicine and Psychotherapy, University of Dresden Medical School, Dresden, Germany

## Abstract

As the number of freight trains on railway networks increases, so does the potential for vibration exposure in dwellings nearby to freight railway lines. Nocturnal trains in particular are of particular importance since night-time exposure may interfere with sleep. The present work investigates the impact of vibration and noise from night-time freight trains on human sleep. In an experimental polysomnographic laboratory study, 24 young healthy volunteers with normal hearing were exposed to simulated freight pass-bys with vibration amplitudes of 0.7 and 1.4 mm/s either 20 or 36 times during the night. Stronger vibrations were associated with higher probabilities of event-related arousals and awakenings (p < 0.001), and sleep stage changes (p < 0.05). Sleep macrostructure was most affected in high vibration nights with 36 events, with increased wakefulness (p < 0.05), reduced continual slow wave sleep (p < 0.05), earlier awakenings (p < 0.05) and an overall increase in sleep stage changes (p < 0.05). Subjects reported sleep disturbance due to vibration (F(4,92) = 25.9, p < 0.001) and noise (F(4,92) = 25.9, p < 0.001), with the number of trains having an effect only for the 0.7 mm/s condition (p < 0.05). The findings show that combined vibration and noise from railway freight affects the natural rhythm of sleep, but extrapolation of significance for health outcomes should be approached with caution.

Sleep is a vital biological process which is found, in one form or another, throughout the animal kingdom^1^. Despite the ubiquitous nature of sleep, its core function remains elusive^2^, yet it is recognised as being critical for cognition and physiological function^3^,^4^,^5^,^6^,^7^,^8^. Disturbed sleep has been demonstrated to be associated with multiple negative health outcomes^9^, with include amongst others increased risk for developing type 2 diabetes^10^, adversely impacting on memory consolidation^5^, mood^11^, metabolic and endocrine functions^12^, waking neurobehavioral functions^13^ and creativity^14^, and sleep deprived individuals even superficially appear less healthy and attractive than their well-rested counterparts^15^. Given the importance of sleep, its disturbance by noise is recognised as the most serious non-auditory effect of environmental noise exposure^16^.

Transportation from road, rail and air is the most prevalent source of environmental noise in the home, and a number of previous studies have examined the resulting effects on sleep^17^. Railway noise has been found to have stronger physiological effects on sleep than road and air traffic noise^18^,^19^,^20^ and, in some research, results in a greater percentage of people reporting being highly sleep disturbed than the same levels of road noise^21^. Noise exposure from railways is therefore of concern. Additionally, the environmental policies adopted by many European nations is seeing an increase in the volume of goods transported on the railway networks^22^. Much of this growth is enabled through nocturnal scheduling, with increased levels of night time noise at nearby dwellings a direct consequence. Railway noise exposure has been found to cause self-reported sleep disturbance^23^,^24^, induce awakenings, arousals and cardiac activations^19^, reduce rapid eye movement (REM) sleep and increase wakefulness^18^, increase the probability of sleep medicine intake and actimetry-determined body movements during sleep^18^,^25^, impair daytime attentional processes^26^, and has recently been linked with the risk for certain types of breast cancer^27^. Furthermore, freight train noise is particularly deleterious, and causes more frequent awakenings^28^,^29^, stronger cardiac response^30^ and greater night time annoyance than passenger trains^31^.

The distinct issue of the impact of night time railway freight may be further compounded by strong ground vibrations that often accompany the passage of the heavy freight trains. This can be particularly problematic with poor wheel or rail maintenance, or regions with ground conditions conducive to propagation, with ground vibrations near railway lines commonly in the range of 0.4–1.5 mm/s^32^. Increasing vibration results in increased reporting of waking during the night and waking too early in the morning^33^, and annoyance from railway vibration is higher during the evening than the day, and higher during night than evening^34^. Following nights with vibration exposure of 1.4 mm/s, lower sleep quality and alertness were reported compared to nights with vibration of 0.4 mm/s. This corresponded to higher restlessness, greater difficulty falling asleep and more awakenings during the night^35^. Additionally, stronger vibrations from railway freight are associated reduced subjective sleep quality and increased self-reported sleep disturbance^36^. However, such self-reported sleep variables do not necessarily correlate with actual physiological response^37^, and even minor sleep disturbances which might not contribute to subjective response can result in degradation of executive functions^38^. In addition to exposure level *per se*, there is some evidence that the number of trains influences human response. In epidemiological studies annoyance was found to be higher in areas with a greater number of trains^32^,^39^. Pass-by frequency during the night has furthermore been linked to self-reported noise-induced disturbances^24^. However, the number of nocturnal pass-bys was not found to be statistically significantly associated with polysomnographically measured wakefulness and light sleep time^18^.

To our knowledge, only three studies have previously investigated the physiological effects of vibration on sleep. The first found that simulated vibration from heavy road traffic elicited changes in sleep structure and a decrease in REM sleep^40^. The second and third found that increasing levels of freight train vibration resulted in greater changes of cardiac activity^36^,^41^. However, the question of the impact of vibration and noise from railway freight on sleep structure presently remains unanswered. Therefore, the present study aimed at determining the physiological effects of combined vibration and noise from nocturnal railway freight on sleep. The hypothesis was that sleep disruption would increase both with higher vibration amplitudes and the number of trains during the night.

## Methods

### Factorial study design

The study was a 2 × 2 factorial experiment consisting of two vibration amplitudes (moderate or high maximum acceleration) and two train frequency scenarios (20 or 36 trains) during an 8-hour night time measurement period (23:00–07:00). These 4 exposure nights were preceded by an exposure-free single night for habituation and an exposure-free single control night to measure baseline normal sleep. The 4 exposure nights were presented in a Latin square design over 8 study weeks.

### Experimental procedure

The experimental laboratory is built up as an apartment with three identical bedrooms of a “room-within-room” design. Each participant had access to a private bedroom furnished with a bed, desk, chairs, bedside cabinet and lamps and to a living and kitchen area, Participants arrived at the laboratory by 20:00 each evening to allow sufficient time for electrode placement, and also to ensure they were relaxed by lights-out at 23:00. They were informed during an orientation procedure that the trial aim was to investigate train exposure but they were blind to the exposures over experimental nights.

An initial exposure-free habituation night allowed time for adaptation to the setting and measurement apparatus, and this night was not included in the analysis. The second night was again an exposure-free condition, serving for baseline measurement of normal sleep (control night). The 4 subsequent nights served as exposure conditions during which vibration and noise from freight passages was presented (see Table 1).

### Vibration and noise exposure

**Figure 1:**
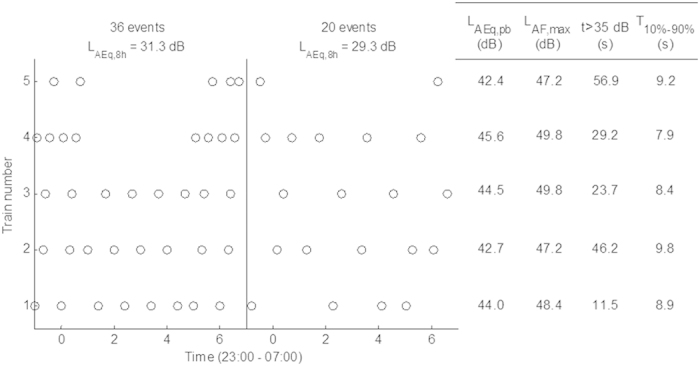
Visual representation of train distribution in 36 event night (left) and 20 event night (centre). Acoustic characteristics of the auditory stimuli (right). L_AEq,8h_ = 8 hour equivalent noise level. L_AEq.pb_ = Pass-by noise level. L_AF,max_ = Maximum noise level, fast time constant. t > 35 dB = pass-by duration. T_10–90%_ = noise rise time. Noise levels are measured at the pillow position.

Exposure nights were constructed from a pool of 5 individual stimuli, the synthesis of which is described in detail elsewhere^36^ and only summarized here. Five audio recordings of freight train pass-bys were low-pass filtered to correspond to a closed window based on the curve of reference values for airborne sound as defined in ISO 717-1:1997^42^. The characteristics of each train are presented in Fig. 1. The low frequencies (<125 Hz) of the train noise was introduced via eighty eight 10” loudspeakers concealed within the ceiling of each bedroom. The higher frequency components were reproduced by loudspeaker cabinets in two of the upper corners of each room. Noise and vibration exposure was introduced using a fully automated system from a separate control room. Since the background levels in the bedrooms were unnaturally low (<14dBA), band-pass filtered pink noise simulating ventilation noise was introduced throughout the trial at a level of 25 dBA measured at the pillow. Each stimulus was accompanied by vibration (rise time (0 mm/s to first peak) = 5.6 s). A 10 Hz sinusoid was amplitude modulated using *c*(*t*) given in equation (Equation 1). The modulation introduces sidebands around the main 10 Hz component from 8.3 to 11.7 Hz, but the secondary peaks are 15 to 25 dB lower than the carrier signal. The signal was generated after inspecting many indoor measurements and forming a repeating simple signal with the same time characteristics as a typical measurement^32^,^43^. The shape of the waveform was verified by measurements on the frame of the bed used in the lab artificially loaded with weights of 75 kg. Amplitude *A* was adjusted such that the maximum root mean square (rms) W_d_ weighted acceleration^44^ was either 0.0204 ms^−2^ (“high”) or 0.0102 ms^−2^ (“moderate”). Perception thresholds are almost identical for recumbent persons along either the head-foot or side-side axis, only differing slightly at frequencies below 4 Hz and above 31.5 Hz^45^. The bed frame was therefore excited horizontally along the lengthwise axis^36^ by electrodynamic transducers (Quake Q10B, frequency response 5–40 Hz). Each shaker was driven separately by a 1000 W power amplifier (BKA1000-4A).


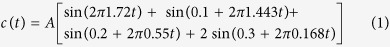


Either 20 or 36 trains were used in each exposure night, with all events within nights having the same maximum vibration. The resulting conditions are therefore 20 trains, noise + moderate vibration (NVm20), 20 trains, noise + high vibration (NVh20), 36 trains, noise + moderate vibration (NVm36) and 36 trains, noise + high vibration (NVh36), see Table 1. Typical night-time freight timetabling scenarios were reproduced by having a greater train frequency in the first two and final two hours of the night (see Fig. 1). The 8 hour vibration exposures are presented as rms (Equation 2) in Table 1. *a*_*w*_(*t*) is the weighted (*w*) acceleration as a function of time and *T* is the duration of the measurement period. Vibration velocity is reported using comfort weighting^46^, which is the standard used in the Scandinavian countries, and is similar to the W_m_ weighting described in ISO 2631-2:2003^47^.


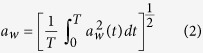


### Questionnaires

Participants completed a questionnaire each morning immediately following the alarm call. Five-category Likert scales were used to determine sleep quality (*Very good, Good, Not particularly good, Poor, Very poor*), sleep disturbance due to either noise or vibration (*Not at all*, *Slightly*, *Rather*, *Very* and *Extremely*) and whether each stimulus disturbed their sleep such that they slept poorly, awoke, had difficulty falling asleep and caused morning tiredness (*Not at all, Slightly, Rather, Very, Extremely*). Eleven-point numerical scales were used to record sleep quality (anchor points *Very good* and *Very bad*), morning restoration (*Very rested*-*Very tired*, *Very relaxed*-*Very tense* and *Very irritated*-*Very glad*) and self-assessment of sleep *(Easy/difficult to fall asleep, Slept better/worse than usual, Deep/shallow sleep, Never/Often woke*). Within the 15 minutes preceding lights-out each night, participants completed a separate questionnaire with the same questions on tiredness, alertness, mood and stress/energy.

### Polysomnography

Objective sleep variables were obtained using PSG. All electrode placements and sampling and filter frequencies were as per current standards^48^.

The recorded PSG data were blindly scored by a trained sleep technician according to the current AASM standard^48^. Identification of EEG arousals was performed in accordance with relevant guidance^49^. Arousals of >15 s duration were classed as awakenings.

Sleep onset latency (SOL) was defined as time until the first occurrence of a non-wake epoch after lights out. Rapid eye movement (REM) sleep and slow wave sleep (SWS) latency were the first occurrences of stage REM and N3 respectively following sleep onset. Sleep period time (SPT) was the time from sleep onset to final awakening. Wakefulness after sleep onset (WASO) was the time spent in stage W after sleep onset. Total sleep time (TST) was SPT minus WASO. Sleep efficiency was the ratio of TST to time in bed (TIB, 480 minutes). Sleep stage changes (SSCs) were defined as moving from one sleep stage to a ‘lighter’ stage. Changes to stage W were not considered as SSCs but treated separately as awakenings. REM was classified as the ‘lightest’ stage in accordance with Carter *et al*^50^. and so a SSC cannot occur from REM. Possible changes were therefore N1 to REM, N2 to REM or N1, and N3 to REM, N1 or N2. The increased likelihood, relative to spontaneously occurring reactions, of an arousal, awakening or SSC occurring following the start of a train was determined for all exposure conditions. The derivation of these event-related probabilities is presented in the Supplemental Methods.

### Study subjects

Twenty-four volunteers were recruited via public advertising (13 females 11 males, age range 19–28, mean 22.9 s.d.±2.8 years). Participants were required to maintain normal sleeping patterns determined via self-report, have good hearing (≤20 dB HL, 250 Hz–8 kHz, screened using pure tone audiometry prior to acceptance) and not use tobacco products or take regular medication affecting sleep. To avoid potential breathing difficulties or apnoea they were required to have a BMI within the normal range of 18.5–24.99. They were prohibited from alcohol throughout the study, caffeine consumption after 15:00 and sleeping at times other than the exposure period of 23:00–07:00.

The study followed the Declaration of Helsinki on Biomedical Research Involving Human Subjects and was approved by the Ethics Committee from the University of Gothenburg. Volunteers were financially compensated for participation and provided informed written consent prior to commencement of the study.

### Statistical Analysis

Questionnaire data were analysed using repeated measurement ANOVA (IBM SPSS v. 18, USA). The control night was included in the analysis when examining whether noise and vibration exposure had an effect relative to baseline normal sleep. The four exposure nights alone (i.e. excluding control) were included in the analysis when investigating effects of vibration amplitude or number of events. To account for multiple testing, Bonferroni corrections were used in the questionnaire data analysis.

PSG data were analysed in SAS (v. 9.4 Cary, NC, USA). Variables describing sleep macrostructure were analysed with respect to the five different study nights. Mixed models were used to account for correlation between measurements on the same individual. Gender was included in the model as an explanatory factor. For each polysomnogram outcome, p-values for pairwise comparisons were corrected for multiple testing using Tukey adjustments. A detailed description of the approach used in the statistical model is presented in the Supplementary Methods.

## Results

Due to a technical issue, PSG recordings were not obtained for a single female participant in the NVh20 condition.

### Polysomnogram data

#### Sleep macrostructure

Sleep macrostructure data are presented in Table 2. Relative to the control, the total number of changes to lighter sleep stages was significantly greater in the NVh36 condition (p < 0.05). The first awakening after sleep onset occurred earlier in NVh36 condition (p < 0.05), the difference to the control night being 25.3 minutes. The maximum length of uninterrupted time spent in N3 was on average 5.6 minutes shorter in NVh36 as compared to the control (p < 0.05). In high vibration nights, WASO increased by 7.2 minutes with the increased number of trains (p < 0.05). Regarding gender, only the number of arousals was found to differ (p = 0.025), with males having more arousals averaged over all 5 nights (59.9) than females (46.4).

#### Sleep microstructure

**Figure 2:**
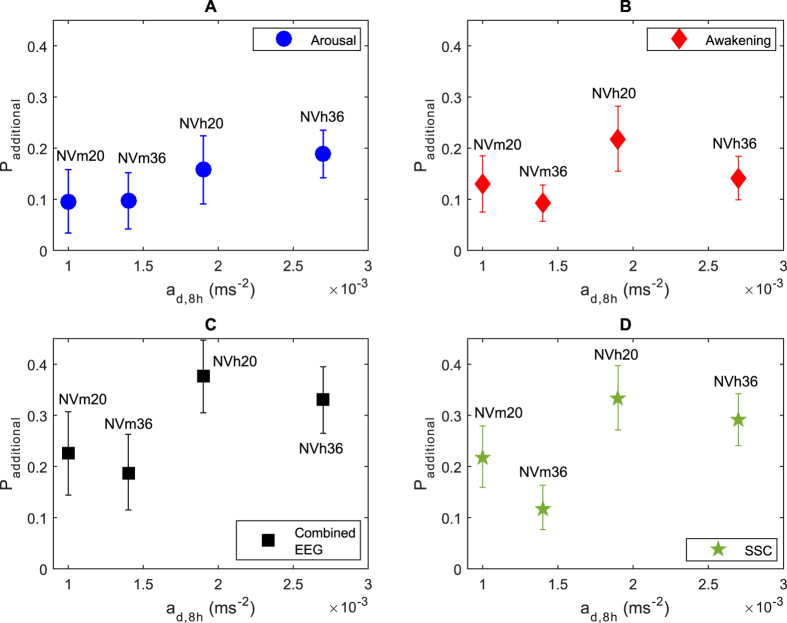
Additional probabilities and 95% confidence intervals of arousals (**A**), awakenings (**B**), combined EEG arousals and awakenings (**C**) and sleep stage changes (**D**) in the experimental nights.

Applying a 60 s analysis window (see Supplementary Results for rationale), the probability of observing combined EEG reactions (P_EEG,ob_ p < 0.0001) and sleep stage changes (P_SSC,ob_ p < 0.0001) was significantly higher in all exposure nights than in the control night. Figure 2 presents the additional probabilities relative to the baseline as determined in the control condition for arousals (Fig. 2A), awakenings (Fig. 2B), combined EEG reactions (Fig. 2C) and SSCs (Fig. 2D) over the full course of the night. P_SSC.additional_ and P_EEG.additional_ were greater in high vibration nights compared to moderate vibration nights with the same event distribution (p < 0.001 for EEG, see Fig. 2C, p < 0.05 for SSCs, see Fig. 2D). In nights with moderate vibration, P_SSC.additional_ was lower in the 36 event night than the 20 event night. No other significant effects were observed between nights with different numbers of trains.

**Figure 3:**
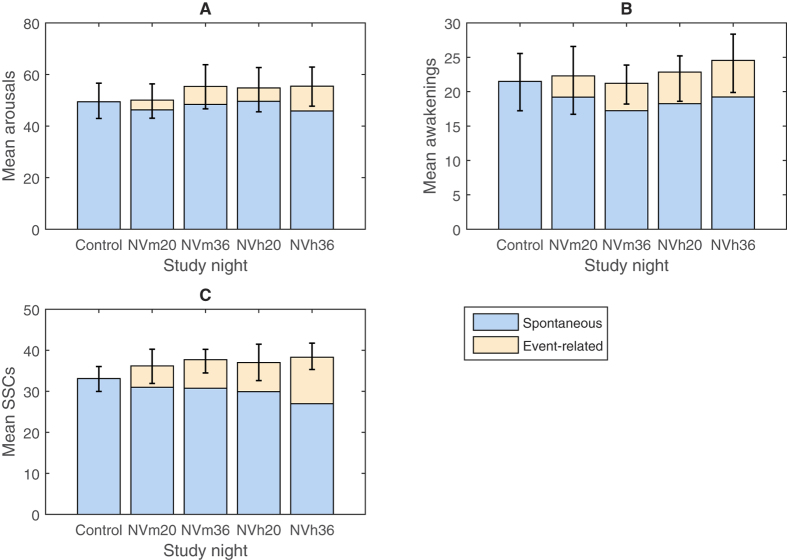
Mean number of spontaneous and event related arousals (**A**), awakenings (**B**) and sleep stage changes (**C**) in all experimental nights. Error bars indicate 95% confidence intervals.

The mean arousals, awakenings and SSCs occurring within each night are presented in Fig. 3. All reactions in the control night happened in the absence of any exposure and are all therefore spontaneous. In the exposure nights, reactions occurring within the analysed 60 s time windows have been considered to be ‘event-related’, i.e. those used in the derivation of P_additional_. Reactions occurring outside of these analysis windows have been classed as spontaneous. There were more SSCs in high vibration nights with 36 trains relative to the control (see Table 2). From Fig. 3 can be seen that the number of spontaneous reactions generally seemed to decrease and that event-related SSCs occurred at the expense of these spontaneous reactions. For arousals and awakenings, there was no significant increase in the total number of responses in exposure nights, although event-related reactions generally seemed to occur at the expense of spontaneous reactions similarly to the effect seen with SSCs.

### Self-reported questionnaire data

**Figure 4:**
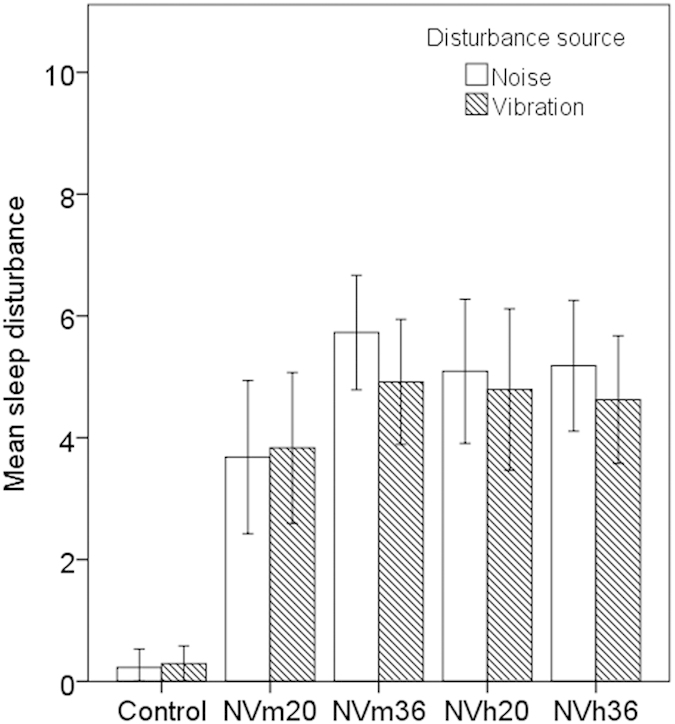
Mean rating of self-reported sleep disturbance from noise and vibration during the exposure nights, recorded immediately after awakening. Error bars indicate 95% confidence intervals.

Results from the morning questionnaires are presented in Supplementary Table 1. No effects of exposure night presentation ordering were found. The exposure conditions had an impact on sleep disturbance from vibrations (F(4, 92) = 25.9, p < 0.001, Fig. 4). Participants reported higher sleep disturbance by vibration in all exposure nights as compared to the control (p < 0.01). In nights with moderate vibration there was an effect of the number of trains (p < 0.05), with participants being more disturbed by vibration in exposure conditions with a higher number of events.

A significant effect of exposure was also found for sleep disturbance from noise (F(4, 84) = 37.9, p < 0.01, Fig. 4). For all experimental nights, participants reported higher disturbance by noise compared to the control (p < 0.01). Additionally there was an effect of number of trains on disturbance in nights with moderate vibration, (p < 0.05), with higher disturbance by noise for the higher number of trains.

No significant effect of exposure condition was observed for sleep quality, the morning restoration questions (rested, at ease, irritated, never woke), the stress-energy or mood scales.

There were no significant effects of exposure on the evening questions. No significant main effects of exposure or interactions between morning and evening questionnaires for the stress-energy or the mood items were found.

## Discussion

This study presents for the first time polysomnographically evaluated effects on sleep due to railway vibration. It expands upon the limited existing work^40^,^51^ investigating the sleep impact of vibration by both incorporating physiological measures of sleep, and improving the exposures used. We hypothesised that stronger maximal vibration would impact on sleep, including global sleep structure and event-related reactions. The study shows a clear influence of vibration on SSC and EEG response, with a moderate effect on overall sleep macrostructure and subjective outcomes.

### Effects of vibration amplitude

In all exposure nights, the additional probability of arousals or awakenings was higher than the likelihood of them occurring spontaneously as part of the natural rhythm of sleep. Reaction probabilities were higher in NVh36 versus NVm36 and in NVh20 versus NVm20. The noise exposure and the distribution of trains were identical in both of the 36 event nights and both the 20 event nights. The only change in exposure was an increase of vibration amplitude, from moderate to high. Thus the increased vibration amplitude was associated with increased probability of arousals and awakenings. Awakenings and arousals can be viewed as short-term acute effects, and the health impact of such acute reactions may in the short-term be small. However, arousals and awakenings in particular are good indicators of fragmented sleep^52^, and noise-induced sleep fragmentation may in the long run manifest as chronic conditions such as cardiovascular diseases^53^ and metabolic illness^54^. Studies in mice indicate that the restorative function of sleep may arise from the brain switching to a state allowing the removal of neural waste products that accumulate during wakefulness^4^. The disruption of natural sleep rhythms due to vibration from railway freight traffic as observed in the present work might also interfere with such processes.

In addition to the increased probability of arousals and awakenings following higher vibration amplitudes, higher probabilities of SSCs were found when increasing vibration amplitudes from moderate to high for the same number of trains. The presence of strong vibrations thus had a greater impact on alterations of sleep depth. Despite there being no consensus on the relative functions of the various sleep stages, there is converging evidence that central neural processes during sleep play an important role in memory consolidation^5^. Slow wave sleep in particular has been identified as important for declarative memory in humans, through mechanisms involving offline replay of spatial tasks in the hippocampus^55^. Furthermore, SWS is considered to be important for physical restoration^56^ and is accordingly prioritized after sleep deprivation^57^, while REM sleep is believed to be important for cognition^58^. Forced changes of sleep structure by strong vibration altering the normal shifts of sleep stage could therefore contribute towards disruption of physical and mental restoration.

No increase in the total number of arousals, awakenings or SSCs was observed in any of the exposure nights, with the single exception of there being 5.2 more SSCs in the NVh36 night than the control. However, the additional probability of either an arousal or awakening was around 15% in nights with moderate vibration, and around 30% following high vibrations. In general, these event-related arousals, awakenings and SSCs did not occur in addition to reactions that happen as part of the natural sleep process. Instead these event-related reactions are replacing those that would have occurred spontaneously, in the absence of exposure. This supports previous work showing that traffic noise-induced arousals and awakenings occur at the expense of reactions that would have otherwise occurred spontaneously throughout the night^19^. To our knowledge, the present work is the first time this indicated redistribution is also valid for vibration, and the first time it has been shown at all for SSCs from any traffic exposure. Although efforts were made in the calculation of window length to maximize the likelihood that reactions were a direct result of the exposure stimuli, it cannot be totally excluded that reactions occurring within the window are not spontaneous. On the other hand, neither can it be said with certainty that reactions outside of the window are not caused by the vibration and noise.

### Effects of number of trains

A number of effects due to differences in the number of trains were seen. In nights with moderate vibration, self-reported disturbance by both vibration and noise was higher when increasing the number of trains from 20 to 36, eliciting disturbance levels similar to those seen in the high vibration conditions. Although no corresponding differences in the sleep macrostructure were found between moderate vibration nights with a higher number of trains, the mean number of event-related awakenings almost doubled from 2.3 to 3.9. In the high vibration nights with 20 and 36 trains, the mean numbers of event-related awakenings were 3.7 and 5.0 respectively, corresponding to an increase in wakefulness (WASO) of 7.2 minutes. These high vibration condition event-related awakenings are rather similar to 3.9 in the NVm36 condition. Should a participant awake as a consequence of train event, and recall the awakening the following morning, it is reasonable that they would ascribe the train vibration and/or noise as a source of nocturnal disturbance. If so, an increase in self-reported disturbance might be expected given a greater number of event-related awakenings. Equally, nights with a similar number of event-related awakenings would be anticipated to elicit similar self-reported disturbance to one another, such as is seen here. Support for this was also given by Elmenhorst *et al.*, who found that only the number of nocturnal train events was significantly associated with night-time annoyance^29^.

In general, the number of events had no effect on the probability of an EEG reaction. This contrasts previous work, which has observed a decrease in awakening and arousal probability when doubling the number of noise events per night^19^. Unlike auditory perception, somatosensory tactile perception involves four independent mechanoreceptive systems, and at least two of these are responsible for vibration detection, namely those whose afferents end in either Meissner or Pacinian corpuscles. One explanation for the lack of observed effect for number of events could therefore be that auditory habituation occurs within nights following repeated noise exposure, but this is not necessarily true for the distinct vibration perception system. The mechanisms which might be involved in potential vibration habituation or sensitization effects warrant further future studies.

One exception to the lack of effect of number of trains on event-related reaction probabilities was found. In nights with moderate vibration, event-related SSCs were more likely when there were 20 rather than 36 trains. Subjects reported greater vibration disturbance in the night with 36 trains, which was also the night with the lower SSC probability (NVm36). This seemingly contradictory result could be explained by there being nearly twice the number of event-related awakenings occurring in the NVm36 night. SSC probability does not include awakenings, so exposure to a higher number of trains appears to have resulted in a higher total number of full awakenings, rather than only an alteration of sleep depth. The indication that the number of trains during the night increases subjective sleep disturbance is supported by annoyance data in the field^32^. No differences in subjective sleep quality were observed between nights despite the reporting of sleep disturbance following exposure. This may be explained by the fact that sleep quality is primarily linked with time in SWS and sleep efficiency, neither of which differed across sleep architectures^59^.

Vibration amplitude and the number of trains together both impacted upon a number of sleep macrostructure outcomes in the polysomnogram. Although there were around 5 more SSCs during NVh36 than the control as noted earlier, there were no differences in the time spent in the different sleep stages, meaning that sleep rebounded to the deeper stages rather quickly following disruption. In addition to the greater number of SSCs, participants also awoke for the first time 25.3 minutes earlier, and had a reduction of 5.6 minutes in continuous time in slow wave sleep during NVh36 than in the control. Subtle disruptions of normal sleep rhythm, such as sleep stage changes and the reduced SWS continuity may be important regarding health in the mid- to long-term perspective for exposed populations. As little as one night of SWS suppression has been shown to reduce glucose metabolism the following morning. Over time, such changes might increase the risk of metabolic disorders, including type 2 diabetes mellitus and obesity, although it is unclear what degree of glucose suppression is clinically relevant^60^.

For all cases where an impact on sleep is found, the most deleterious effects are observed during the condition with high vibration and a higher number of trains, providing additional support for the initial hypothesis that the number of trains would contribute towards sleep impairment.

### Influence of noise

Humans, like most organisms, have several distinct sensory mechanisms, yet the inputs of these quite separate channels are able to merge in to a coherent perception of the surrounding environment^61^. However, the interactions between multisensory inputs are not always clear. It has previously been found that the presence of strong vibration increases the annoyance due to railway noise^32^. On the basis of the current work it cannot be concluded whether the increased probabilities for both SSCs and EEG reactions are due to the body reacting to the vibration directly, or if there is a cross-modal effect whereby the occurrence of vibrations lowers the threshold of a reaction due to noise (or vice versa). Such multimodal effects have previously been reported to occur in response to low intensity combined auditory and somatosensory stimulation in monkeys, where neuronal oscillations were found to be synergistic, whereby the response amplitude was greater than the sum of the responses to each individual stimulus^62^. Additional work involving conditions with vibration in the absence of noise and vice versa is required to examine possible interactions.

### Limitations

Only volunteers reporting having good normal sleep were included in the study, and recruited via public advertising. As a result of this self-selection of individuals having robust typical sleeping patterns, it is possible that the study population represented a group particularly impervious to vibration and noise exposure. In the field where the prevalence of sleep problems or disorders may be higher^63^, the response to these exposures might be greater. Conversely, it cannot be excluded that a degree of habituation might occur amongst exposed populations, resulting in some measure of response inhibition. The sample size of the present study may also limit the conclusions that can be drawn regarding the questionnaire data, and it is possible that we have missed identifying low-level effects due to statistical lack of power. Only horizontal vibration was used, and in the field vibration will typically occur along all three orthogonal directions. Although vertical vibration amplitude is often higher than horizontal vibration when measured on a floor mid-span, horizontal vibration increases monotonically with building level^64^. When considering whole body vibration for persons in bed, vertical vibration is greatly attenuated by the mattress, whereas horizontal vibration can be amplified relative to vibration on the bed frame^40^. Horizontal freight vibration often peaks at around 6–8 Hz, and the perception threshold for recumbent persons is most sensitive below 10 Hz^45^. It would therefore have been desirable for the vibration signals to have had a dominant component slightly below the 10 Hz frequency used. However, due to limitations of the equipment and unwanted distortion products below this frequency, it was necessary to use signals with a 10 Hz main component. If reactions would indeed be stronger at lower frequencies, then it is conceivable that the results might slightly underestimate the effects of vibration exposure in the field.

## Conclusions

Vibration and noise from railway freight traffic was found to impact on sleep. The amplitude of vibration contributed towards arousal, awakening and sleep stage change probabilities. The number of trains during the night contributed towards self-reported sleep disturbance at moderate vibration amplitudes. Both vibration amplitude and the number of trains together contributed towards effects on sleep macrostructure, whereby the number of sleep depth changes, slow wave sleep continuity and nocturnal wakefulness were negatively affected during the high vibration condition with a high number of trains. Taken together, the results indicate that vibration and noise affects the natural rhythm of sleep, but extrapolation of significance for health outcomes should be approached with caution. Studies into deleterious long-term effects of nocturnal vibration exposure are lacking, as are studies examining cross-modality between noise and vibration, and provide interesting avenues for future research.

## Additional Information

**How to cite this article**: Smith, M. G. *et al.* Vibration from freight trains fragments sleep: A polysomnographic study. *Sci. Rep.* **6**, 24717; doi: 10.1038/srep24717 (2016).

## Supplementary Material

Supplementary Information

## Figures and Tables

**Table 1:** Eight hour nocturnal exposures.

Experimental night	a_d,max_ (ms^−2^)	Velocity (mms^−1^)	a_d,8h_ (ms^−2^)	L_AEq,8h_ (dB)	L_AF,max_ (dB)
NVm20	0.0102	0.7	0.0010	29.3	49.8
NVm36	0.0102	0.7	0.0014	31.3	49.8
NVh20	0.0204	1.4	0.0019	29.3	49.8
NVh36	0.0204	1.4	0.0027	31.3	49.8

a_d,max_ = maximum d-weighted acceleration, 1 s time constant. Velocity = maximum velocity (comfort weighted), 1 s time constant. a_d,8h_ = 8 hour (23:00–07:00) d-weighted acceleration. L_AEq,8h_ = 8 hour (23:00–07:00) equivalent A-weighted sound pressure level. L_AF,max_ = Maximum A-weighted sound pressure level (fast time constant). Vibration values are measured on the bed frame. Noise levels are measured at the pillow position.

**Table 2:** Means and standard deviations for polysomnographically determined sleep macrostructure parameters across all experiment nights.

Variable	ControlMean ± SD	NVm20Mean ± SD	NVh20Mean ± SD	NVm36Mean ± SD	NVh36Mean ± SD
Sleep latency (min)	26.4 ± 19.9	16.6 ± 14.7	22.3 ± 18.2	17.4 ± 10.5	15.3 ± 14.0
REM latency (min)	80.7 ± 19.2	81.0 ± 36.3	87.9 ± 33.4	84.9 ± 35.2	96.0 ± 49.1
N3 latency (min)	20.0 ± 18.6	18.2 ± 9.7	20.5 ± 13.2	19.4 ± 6.5	23.1 ± 13.6
Total SSCs (n)	33.1∗ ± 6.8	36.2 ± 9.2	37.0 ± 10.0	37.7 ± 6.5	38.3∗ ± 7.2
EEG arousals (n)	49.4 ± 15.6	49.3 ± 15.2	54.1 ± 19.9	55.3 ± 19.3	55.1 ± 17.2
EEG awakenings (n)	21.5 ± 9.4	21.5 ± 11.2	21.9 ± 7.6	21.1 ± 6.4	24.1 ± 9.6
WASO (min)	21.0 ± 14.2	28.3 ± 38.3	19.2∗ ± 12.2	22.3 ± 16.6	26.4∗ ± 19.4
First awakening (min)	39.8∗ ± 49.3	16.7 ± 25.9	27.2 ± 38.1	28.8 ± 42.4	14.5∗ ± 35.9
Max. uninterrupted REM duration (min)	20.7 ± 9.2	17.1 ± 6.6	17.9 ± 5.6	20.2 ± 9.6	20.1 ± 8.1
Max. uninterrupted N3 duration (min)	32.0∗ ± 10.6	31.2 ± 10.7	28.8 ± 10.0	32.3 ± 9.8	26.4∗ ± 11.0
EEG arousals per hour of TST (n)	6.9 ± 2.1	6.8 ± 2.0	7.4 ± 2.6	7.5 ± 2.5	7.5 ± 2.3
EEG awakenings per hour of TST (n)	3.0 ± 1.4	3.0 ± 1.7	3.0 ± 1.1	2.9 ± 0.9	3.3 ± 1.4
Sleep efficiency (%)	90.1 ± 5.5	90.7 ± 9.3	91.4 ± 4.6	91.4 ± 4.8	91.3 ± 5.3
Time in N1 (min)	42.6 ± 15.6	42.6 ± 14.9	42.2 ± 14.3	45.0 ± 13.3	48.7 ± 14.7
Time in N2 (min)	212.0 ± 33.0	211.2 ± 41.3	210.9 ± 31.7	215.0 ± 30.9	224.0 ± 26.7
Time in N3 (min)	90.0 ± 25.8	91.3 ± 23.6	94.8 ± 21.0	91.1 ± 25.9	84.4 ± 21.6
Time in REM (min)	88.0 ± 19.3	90.0 ± 25.5	90.7 ± 17.8	87.6 ± 21.0	81.4 ± 18.9

Significant pairwise comparisons are indicated with ^∗^and underlined. NVm20 = Noise and moderate vibration (0.7 mm/s), 20 trains; NVm36 = Noise and moderate vibration (0.7 mm/s), 36 trains; NVh20 = Noise and high vibration (1.4 mm/s), 20 trains; NVh36 = Noise and high vibration (1.4 mm/s), 36 trains.
