# Supplementary material for: Vibration from freight trains fragments sleep: A polysomnographic study
Source: Sci Rep. 2016 Apr 19;6:24717. doi: 10.1038/srep24717 (PMC4835753; doi:10.1038/srep24717)
Supplement: Supplementary Information [file srep24717-s1.pdf]

Supplementary material for "Vibration from freight trains fragments sleep: A polysomnographic study".

Michael G. Smith[1]*, Ilona Croy[2], Oscar Hammar[1], Kerstin Persson Waye[1]

[1] Department of Occupational and Environmental Medicine, The Sahlgrenska Academy at the University of Gothenburg, Gothenburg, Sweden

[2] Department of Psychosomatic Medicine and Psychotherapy, University of Dresden Medical School, Dresden, Germany

Supplementary Methods

Polysomnography

Electroencephalogram (EEG), left and right electrooculogram (EOG) and submental electromyogram (EMG) were recorded using surface electrodes onto an ambulatory PSG device (SOMNOscreen plus PSG+, SOMNOmedics GmbH, Germany) at recommended sampling and filter frequencies.[1] Frontal, central, occipital and mastoid EEG electrodes were affixed according to the international 10-20 system. Surface impedance values were ≤5kΩ following the placement procedure. A finger pulse oximeter was used to record saturation of peripheral oxygen (S_pO_2), finger pulse rate and plethysmogram data. Breathing rate and amplitude was monitored using two piezoelectric respiratory effort belts. Participants were also fitted with two torso electrocardiograph electrodes to record cardiac activity, the results of which are reported elsewhere.[2]

Event-related probabilities

The probabilities of EEG arousals, EEG awakenings, and SSCs were determined by examining whether or not they occur within a number of time windows over the course of a night. The probabilities of *observing* either an arousal, $P_{Ar,ob}$, an awakening $P_{Aw,ob}$, or either of each of these EEG reactions, $P_{EEG,ob}$ in these time windows on a given night are given by equations 3-5. The probability of observing a sleep stage change, $P_{SSC,ob}$, is given by equation 6. Ar_{ob}= number of time windows containing at least one arousal, Aw_{ob} number of time windows containing at least one awakening, and SSC_{ob}=number of time windows containing at least one sleep stage change. N=number of trains during the night (20 or 36), W_n=number of events where the baseline epoch preceding the noise window (from here on termed NE0) is scored as Wake and R_n=number of events where NE0 is scored as REM. Therefore, any time windows where the participant was already awake are excluded from analysis.

$$P_{Ar,ob} = \frac{Ar_{ob}}{N - W_n} \tag{S1}$$

$$P_{Aw,ob} = \frac{Aw_{ob}}{N - W_n} \tag{S2}$$

$$P_{EEG,ob} = \frac{Ar_{ob} + Aw_{ob}}{N - W_n} = P_{Ar,ob} + P_{Aw,ob} \qquad \text{(S3)}$$

$$P_{SSC,ob} = \frac{SSC_{ob}}{N - W_n - R_n} \qquad \text{(S4)}$$

The first 30s epoch containing >15s of noise and vibration for each individual event was classified as “Noise Epoch 1” (NE1). NE1 immediately follows NE0 in time. NE1 is followed by subsequent 30s noise epochs, the number of which is set by the length of the analysis window chosen, e.g. a 60s time window will have 2 noise epochs, NE1 and NE2. These epochs were compared to NE0 to determine whether a SSC has occurred.

Arousals, awakenings and sleep stage changes occur spontaneously as part of the natural rhythm of sleep. The probability of these *spontaneous* reactions is determined by analysing 36 exposure-free time windows in the control night for each participant. The spontaneous probability of arousals, $P_{Ar,sp}$, awakenings, $P_{aw,sp}$, a combination of both arousals and awakenings, $P_{EEG,sp}$, and sleep stage changes, $P_{SSC,sp}$ are therefore given by applying equations 3-6 to the control night.

Of interest here are a participant’s *additional* probability of a vibration and noise event leading to arousals, awakenings and SSCs. These are defined as the probability of at least one of the reactions of interest occurring within the time window, but when no concurrent spontaneous reactions would have occurred in the same window.[3] The reaction is therefore due to the event alone. This additional probability, $P_{additional}$, is given by equation 7, where the subscripts *react* refer to the reaction of interest, either an arousal *Ar*, awakening *Aw*, combined EEG reaction *EEG*, or sleep stage change *SSC*.

$$P_{react,additional} = P_{react,ob} - P_{react,sp} \qquad \text{(S5)}$$

Both observed and spontaneous probabilities are influenced by the length of the time window chosen. This should be as long as necessary for a train event to fully exploit its reaction potential.[3] In other words, the window duration should be that which gives $P_{additional}$ a maximal value, and can be determined empirically using different window lengths.

Statistics – Polysomnogram analysis

Macrostructure variables were analysed by ensuring normal distribution following appropriate transformations. The residuals from each analysis of log transform, square root transform and raw value were visually inspected for departure from the normality assumption and the transform with the least departure was chosen for each outcome. Two different structures were considered for the correlations between measurements from the same individual. The simpler compound symmetry structure assumes the same correlation for all pairs of observations from the same individual and was used unless this structure was rejected by a likelihood test (α=0.05). If this simple structure was rejected, then an unstructured covariance matrix was assumed. This structure allows for different correlations for different pairs of measurements from the same individual.

Binomial distribution was assumed for event-related probabilities. If data showed more variance than predicted by binomial distribution, then an overdispersion parameter was used to correctly assess the standard errors.

Supplementary Results

A. Analysis time window

Sleep stage changes and awakenings were scored based on a 30s epoch length, so therefore the duration of the analysis window must be an integer multiple of 30s. The additional probability of observing an awakening or arousal compared to the spontaneous reaction probability, $P_{EEG,additional}$ (see S(S3 and S(S5) was calculated for all exposure nights using window lengths of 30, 60, 90, 120 and 150s. The effect of time window length on $P_{EEG,additional}$ is shown in Figure S1A, along with the mean probability determined from all exposure nights. The mean additional probability is maximal with a 60s window ($P_{EEG,additional}$=0.255), therefore all analyses utilise a 60s time window. The probability of spontaneously occurring arousals or awakenings was not dependent on the number or timing of the sampling periods (Figure S1B, paired sample t-test 60s window p=0.583).

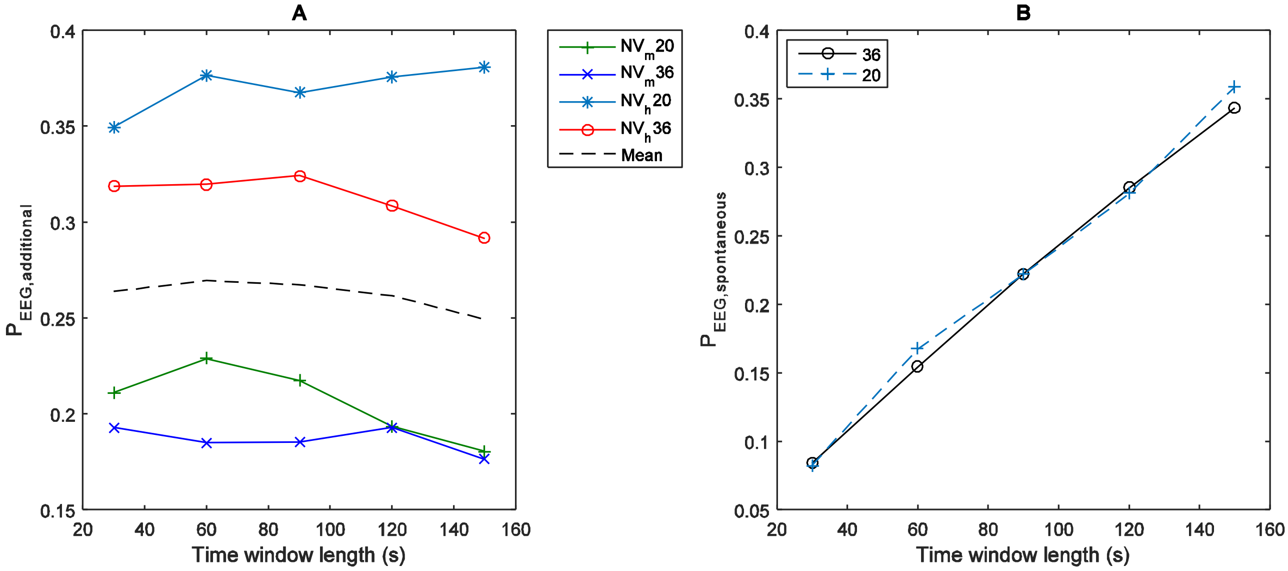

Figure S1 A. Empirically determined effect of length of time window on probability $P_{EEG,additional}$ of either EEG arousal or awakening being attributable to a noise event. B. Spontaneous EEG arousal or awakening probability in the quiet control night, determined using time points corresponding to train times in the 36 and 20 event exposure nights.

Supplementary references

1 Iber, C., Ancoli-Israel, S., Chesson, A. & Quan, S. F. *The AASM Manual for the Scoring of Sleep and Associated Events; Rules, Terminology and Technical Specifications*. 1 edn, (American Academy of Sleep Medicine, 2007).

2 Croy, I., Smith, M. G. & Waye, K. P. Effects of train noise and vibration on human heart rate during sleep: an experimental study. *BMJ Open* **3**, e002655 (2013).

3 Brink, M. *et al.* Determining physiological reaction probabilities to noise events during sleep. *Somnologie* **13**, 236-243 (2009).

Supplementary Tables

Supplementary Table 1 Mean (M) and standard deviations (SD) for subjective data from morning questionnaires

		Control		NVm20		NVh20		NVm36		NVh36	
		M	SD	M	SD	M	SD	M	SD	M	SD
Sleep Quality	Self-reported sleep quality (10 = very good, 0 = very bad)	6.0	1.7	6.3	2.5	5.3	2.2	5.4	2.5	5.5	2.1
Nocturnal restoration	Rested – Tired (0 = rested, 10 = very tired)	4.6	1.9	4.5	2.0	5.0	2.4	5.8	2.1	5.0	2.3
	At ease – Tense (0 = very at ease, 10 = very tense)	3.7	1.9	4.2	2.0	4.1	1.6	4.3	1.6	4.0	1.7
	Glad- Irritated (0 = very glad, 10 = very irritated)	3.5	1.8	3.3	1.8	4.4	2.5	4.0	1.9	4.3	2.0
Sleep parameter	Time to fall asleep (min)	37.5	47.6	24.8	11.7	31.0	18.8	28.5	16.7	30.4	21.2
	Estimated number of wakeups (n)	3.8	3.7	3.1	2.5	3.5	2.6	3.7	2.5	3.8	2.5
	Difficulty falling back asleep following wakeups (1 = did not wake, 2 = no difficulty, 3 = difficulty)	2.1	0.5	2.2	0.5	2.2	0.6	2.2	0.5	2.3	0.6
	Easy to sleep - Difficulty to sleep (0 = easy, 10 = difficult)	4.3	2.8	4.5	2.5	4.9	3.0	4.3	2.5	4.6	2.7
	Slept better than usual - Slept worse than usual (0 = slept better, 10 = slept worse)	6.3	1.9	5.4	1.9	6.1	2.1	6.3	1.7	6.0	2.1
	Deep sleep - Light sleep (0= deep, 10 = light)	5.2	2.0	6.4	10.5	4.5	2.0	5.3	2.0	5.0	1.9
	Woke never - Woke often (0 = never, 10 = often)	5.7	2.8	5.1	2.6	5.7	2.6	5.9	1.9	5.6	2.0
Disturbance	Disturbance from vibrations (0= not at all disturbed, 10 = extremely disturbed)	0.3	0.7	3.8	2.9	4.8	3.1	4.9	2.4	4.6	2.5
	Vibrations causing poor sleep (1 = not at all, 5 = extremely)	1.1	0.3	1.9	1.0	2.3	1.0	2.3	0.8	2.2	0.9
	Vibrations causing awakenings (1 = not at all, 5 = extremely)	1.0	0.2	2.4	1.2	2.5	1.1	2.4	1.1	2.5	0.9
	Vibrations causing difficulty falling asleep (1 = not at all, 5 = extremely)	1.0	0.2	1.9	1.1	1.9	0.9	2.0	1.0	2.1	1.1
	Vibrations causing tiredness in morning (1 = not at all, 5 = extremely)	1.0	0.2	2.0	0.9	2.3	1.1	2.3	1.0	2.3	0.9
	Disturbance from noise (0= not at all disturbed, 10 = extremely disturbed)	0.2	0.7	3.9	2.8	4.9	2.8	5.4	2.4	5.2	2.4
	Noise causing poor sleep (1 = not at all, 5 = extremely)	1.1	0.3	1.9	1.0	2.2	0.9	2.4	0.9	2.3	1.0
	Noise causing awakenings (1 = not at all, 5 = extremely)	1.1	0.4	2.4	1.1	2.5	1.0	2.8	1.0	2.5	1.0
	Noise causing difficulty falling asleep (1 = not at all, 5 = extremely)	1.0	0.2	1.9	1.2	2.0	1.0	2.4	1.0	2.1	1.0
	Noise causing tiredness in morning (1 = not at all, 5 = extremely)	1.0	0.0	2.1	0.9	2.2	1.0	2.5	0.9	2.4	1.1
Pleasantness and Social Orientation	Pleasantness (1 = minimum, 4 = maximum)	3.1	0.4	3.1	0.4	3.1	0.5	3.0	0.5	3.1	0.5
	Social orientation (1 = minimum, 4 = maximum)	3.1	0.4	3.1	0.5	3.1	0.5	3.2	0.6	3.1	0.4
Stress- Energy	Stress	1.8	0.7	1.9	0.7	1.8	0.8	1.9	0.7	1.8	0.7
	Energy	2.4	0.7	2.3	0.7	2.4	0.7	2.1	0.5	2.4	0.8
